# A review of the latest insights into the mechanism of action of strontium in bone

**DOI:** 10.1016/j.bonr.2020.100273

**Published:** 2020-04-24

**Authors:** Daniella Marx, Alireza Rahimnejad Yazdi, Marcello Papini, Mark Towler

**Affiliations:** a Department of Biomedical Engineering, Ryerson University, Toronto M5B 2K3, Ontario, Canada; b Li Ka Shing Knowledge Institute, St. Michael's Hospital, Toronto M5B 1W8, Ontario, Canada; c Department of Mechanical Engineering, Ryerson University, Toronto M5B 2K3, Ontario, Canada

**Keywords:** Strontium, Bone, Mechanism of action, Medicine, Physiology

## Abstract

Interest in strontium (Sr) has persisted over the last three decades due to its unique mechanism of action: it simultaneously promotes osteoblast function and inhibits osteoclast function. While this mechanism of action is strongly supported by *in vitro* studies and small animal trials, recent large-scale clinical trials have demonstrated that orally administered strontium ranelate (SrRan) may have no anabolic effect on bone formation in humans. Yet, there is a strong correlation between Sr accumulation in bone and reduced fracture risk in post-menopausal women, suggesting Sr acts *via* a purely physiochemical mechanism to enhance bone strength. Conversely, the local administration of Sr with the use of modified biomaterials has been shown to enhance bone growth, osseointegration and bone healing at the bone-implant interface, to a greater degree than Sr-free materials. This review summarizes current knowledge of the main cellular and physiochemical mechanisms that underly Sr’s effect in bone, which center around Sr’s similarity to calcium (Ca). We will also summarize the main controversies in Sr research which cast doubt on the ‘dual-acting mechanism’. Lastly, we will explore the effects of Sr-modified bone-implant materials both *in vitro* and *in vivo*, examining whether Sr may act *via* an alternate mechanism when administered locally.

## 1 Introduction

Strontium (Sr) is an alkali earth metal that was first discovered as a result of lead mining in Scotland in the 18th century (Hope, 1798). It is an abundant trace element in ocean water, ground water and the earth's crust and is naturally occurring in the human diet, with the highest concentrations found in leafy greens (64 mg/kg), grains (18 mg/kg) and seafood (24 mg/kg) (Watts and Howe, 2010; Rosenthal et al., 1970). The physiological role of Sr was first observed in 1870, when it was discovered that it could naturally incorporate into the bones of animals fed small doses of the element. (Papillon, 1870) The observation that Sr was a bone-seeking element like calcium (Ca) led to further research into its effects in other organs. In later years it was found that Sr, like Ca, could affect the contractility of the heart, was able to control parathyroid secretions and stimulate uterine contractions (Dow and Stanbury, 1960).

Between 1945 and 1963, the testing of nuclear weapons led to the contamination of ocean and atmospheric environments with Sr-90, a harmful radioactive isotope of Sr that is produced during fission of plutonium and uranium (Prăvălie, 2014). This led to a surge in research on the metabolism of Sr compared to Ca, since both elements form divalent cations with similar sized ionic radii (1.13 Å compared with 0.99 Å for Ca^2+^ ion) (Alexander et al., 1956). Using Sr isotopes like Sr-85 in double-tracer experiments, it was determined that Ca and Sr are handled very similarly in terms of intestinal absorption, renal reabsorption and skeletal storage, with some biological differences. Sr is transported in the blood *via* binding to serum proteins which normally carry Ca (Olehy et al., 1966) and competes with Ca in the intestine and renal tubules for absorption/ reabsorption (Hendrix et al., 1963; Omdahl and DeLuca, 1972). Toxic Sr doses will therefore lead to disturbances in normal Ca homeostasis, such as hypocalcemia and impaired bone mineralization (Morohashi et al., 1994; Morohashi et al., 1993). In humans consuming a normal diet, 99% of ingested Sr is deposited in bone, leading to the replacement of approximately 0.035% of the Ca present in bone (Watts and Howe, 2010). Consuming toxic doses has been found to cause defective bone mineralization resembling rickets/osteomalacia, with more pronounced effects in animals consuming low Ca diets (Watts and Howe, 2010; Morohashi et al., 1994; Omdahl and DeLuca, 1971). These effects have been linked to the alteration in parathyroid hormone and vitamin D3 levels as well as the direct incorporation of Sr into bone.

Due to its affinity for bone, Sr isotopes have been used in medicine therapeutically for the past half century to treat bone-related illnesses. Sr-89 has been used since the 1940's to treat bone pain in patients with metastatic bone cancer, adjunctive to chemo, radiation and hormonal therapy (Altman and Lee, 1996). Sr-85 has been used to study both Sr and Ca metabolism and has also been a powerful tool in the clinical setting for imaging bone lesions in patients with bone cancer (Rosenthall, 1965). The first therapeutic use of stable Sr (non-radioactive) was in 1952 when it was reported that the administration of Sr lactate to osteoporotic patients could re-mineralize the skeleton when taken along with Ca supplements (Shorr and Carter, 1952). This study found that osteoporotic patients taking Sr had increased bone mass, reduced bone pain and increased mineralization. Later, animal trials found that low doses of Sr chloride stimulated increased bone formation, trabecular volume and reduced bone resorption in healthy animals, and reduced the bone loss seen after estrogen deficiency in ovariectomized animals (Morohashi et al., 1993; Marie et al., 1985; Marie and Hott, 1986). These studies provided strong evidence for the use of Sr as an anti-osteoporotic agent, leading to later clinical testing.

The form of Sr approved for pharmacological use is strontium ranelate (SrRan), which is composed of two stable Sr atoms and ranelic acid and is used for the treatment of osteoporosis. Phase 2 clinical trials were conducted in 2002 and included the PREVention Of early postmenopausal bone loss by Strontium ranelate study (PREVOS) and the STRontium Administration for Treatment of Osteoporosis Study (STRATOS), which were aimed at determining the minimum effective dose for therapeutic use (Reginster et al., 2002; Meunier et al., 2002). Phase 3 clinical trials were conducted in 2004 and 2005 which included the Spinal Osteoporosis Therapeutic Intervention study (SOTI) and the TReatment Of Peripheral OSteoporosis study (TROPOS), which were aimed at determining the effectiveness of Sr at preventing new fractures (Meunier et al., 2004; Reginster et al., 2005). These clinical trials presented overwhelming evidence for SrRan's anti-fracture efficacy and ability to significantly increase bone-mineral density (BMD) in post-menopausal women (Reginster et al., 2002)–(Reginster et al., 2005).

Osteoporosis is a bone disease characterized by high rates of bone turnover, trabecular and cortical bone loss, increased fracture risk and low BMD (Seeman and Delmas, 2006). The high rate of bone turnover occurs due to an increase in bone resorption, which is followed by an increase in bone formation due to the tight coupling between these processes (Seeman and Delmas, 2006; Raisz, 2005). However, bone formation is unable to keep up with resorption, and this imbalance leads to compromised microstructure and bone strength. To combat this, anti-osteoporotic drugs act to either enhance bone formation or decrease bone resorption. The novelty of Sr for the treatment of osteoporosis is that it is proposed to affect both aspects of bone remodelling. The majority of *in vitro* experiments support this dual-acting mechanism. Primary osteoblasts and pre-osteoblast cell lines exposed to relevant doses of Sr exhibit increased cell replication and differentiation (Canalis, 1996)–(Bonnelye et al., 2008). Conversely, osteoclasts exposed to Sr present with reduced resorption, differentiation and cell replication (Bonnelye et al., 2008)–(Peng et al., 2011a), as well as increased apoptosis (Hurtel-Lemaire et al., 2009).

This review describes the mechanisms that have been proposed to explain Sr’s effects in bone, as well as the controversies surrounding Sr research. The cellular mechanisms are centered mainly around the Ca-sensing receptor (CaSR) and its ability to mediate bone cell function and respond to Sr, providing evidence for the dual-acting mechanism. The physiochemical mechanisms have been less widely studied, but describe how Sr is able to affect the intrinsic tissue quality of bone by directly incorporating into bone at multiple levels, including the organic matrix and the hydrated layer surrounding hydroxyapatite (HA) crystals, forming sacrificial bonds and stabilizing hydration shells. The dual-acting mechanism has been questioned in recent years due to inconsistencies in both animal studies and clinical trials, inaccurate BMD measurements and novel clinical trials which question Sr’s anabolic action at pharmacological doses. Despite these controversies, Sr-enriched biomaterials consistently perform better than Sr-free materials both *in vitro* and *in vivo*, in terms of bioactivity, cell proliferation, bone healing and osseointegration (Xue et al., 2006)–(Baier et al., 2013). However, it is difficult to separate the effects that surface contact, local pH changes and the release of other ions such as Ca^2+^, phosphorus (P^5+^) and silicon (Si^4+^) may have on bone cell function at the bone-implant interface.

## 2 Effects of Sr *in vitro*

Sr has been found to stimulate osteoblast function and inhibit osteoclast function *in vitro*. The most studied cellular mechanisms directing this response involve the Ca-sensing receptor (CaSR). Therefore, in this chapter, we will first discuss this receptor and the effects of Ca on bone cells, comparing these intracellular signalling cascades with those observed after Sr treatment. Both Sr and Ca have been found to mediate key cellular functions in osteoblast and osteoclast cells. However, Sr also has an effect on cells which lack the CaSR, and therefore, other cation-sensing receptors/molecular targets will be discussed.

### 2.1 Ca-sensing receptor

The Ca-sensing receptor (CaSR) is a g-protein coupled receptor (GPCR) that is localized to bone cells and has recently been implicated in bone remodelling. It has been known since the 1990's that this receptor plays a critical role in maintaining Ca homeostasis by allowing cells in the parathyroid gland and renal tubules to sense extracellular Ca, regulating parathyroid hormone (PTH) secretion and renal Ca handling (Peacock, 2010). Evidently, mutations in CaSR lead to systemically disordered Ca homeostasis (Raue et al., 2006). Extracellular Ca concentration indirectly effects bone remodelling by altering PTH and vitamin D3 levels; however, recent findings suggest that bone cells can also directly sense Ca levels and respond accordingly. *In vitro* reports provide evidence that osteoblasts, osteoclast precursors and mature osteoclasts express the parathyroid CaSR homolog (Yamaguchi et al., 1998; Kanatani et al., 1999). In these cell types, the CaSR has been shown to control key cellular functions such as cell growth, differentiation and apoptosis in response to Ca^2+^ binding, as well as other divalent cations such as Sr^2+^ (Goltzman and Hendy, 2015; Marie, 2010).

### 2.2 Effects of Sr on osteoblast cells

In order to better understand the effect of Sr on osteoblasts, first we will briefly outline how osteoblasts respond to *Ca.* Osteoblast-directed bone formation is tightly coupled to bone resorption; after a resorption pit is formed, osteoblasts are recruited to fill this pit with secreted matrix (Sims and Martin, 2014). Given this coupling, it is plausible that osteoblasts are exposed to high Ca concentrations in the vicinity following resorption, leading to a paracrine activation of the CaSR. Indeed, high Ca has been shown to promote cell replication and osteogenic gene expression in osteoblasts mediated by CaSR (Yamauchi et al., 2005), since the use of a dominant-negative CaSR construct reduces these positive effects. Cell replication and gene expression are mediated by several intracellular pathways following CaSR agonist binding. In response to Ca, the initial conformational change in the receptor leads to the activation of the g-proteins Gαi and Gαq/11, which activate cellular effectors such as phospholipases C, protein kinase C (PKC) and protein kinase A (PKA) (Brown and MacLeod, 2001). Extracellular Ca is linked to the activation of extracellular signal-regulated kinase (ERK1/2) signalling (Dvorak et al., 2004), Akt signalling (Rybchyn et al., 2011), COX-2 expression (Choudhary et al., 2003), NFATc signalling and Wnt expression (Fromigué et al., 2010) in osteoblast cells, which are crucial transduction pathways for cell replication, differentiation and survival. In this section we will discuss how Sr interacts with these same pathways to promote osteoblast function.

#### 2.2.1 Cell replication

The targeted knockdown of CaSR attenuates Sr-mediated cell replication *in vitro*, suggesting it plays a critical role in Sr’s anabolic effects on osteoblast cells. The cellular pathways that occur in response to Sr-binding are somewhat different than with Ca-stimulation: while Ca rapidly activates ERK1/2 and PKC, Sr was found to have a delayed response, activating these proteins only hours later, with a preference for stimulating PKD. Interestingly, this sustained activation of ERK1/2 has been found to be more important for osteoblastic replication than acute activation (Huang et al., 2001), explaining why the dual action of Ca and Sr potentiate the mitogenic response. Sr has been found to be less potent than Ca at stimulating early second messengers such as inositol phosphate (IP) and intracellular Ca, suggesting the two ions act *via* different cellular pathways following CaSR activation to promote cell replication (Chattopadhyay et al., 2007).

#### 2.2.2 Differentiation and survival

Osteoblasts differentiate from bone-marrow mesenchymal stem cells (MSC's) after they express specific osteogenic genes such as alkaline phosphatase (ALP), bone sialoprotein and osteocalcin (OC) (Peng et al., 2009). This process is associated with the activation and phosphorylation of Runx2, a major transcription factor involved in osteoblast cell fate. Sr treatment upregulates osteogenic gene expression in osteoblasts, which has been found to be dependent on mitogen-activated protein kinase (MAPK) signalling and ERK 1/2 phosphorylation, downstream of rat sarcoma viral oncogene homolog (Ras). The targeted knockdown of Ras using siRNA inhibits Sr-mediated expression of osteogenic markers such as Runx2 (Peng et al., 2009). While Ca stimulation *via* CaSR has also been shown to activate this pathway (Yamaguchi et al., 2000), further research is required to determine if this is mediated by CaSR in response to Sr stimulation. Another pathway involved in osteoblast differentiation is mediated by cyclooxygenase-2 (COX-2), an enzyme that catalyzes the conversion of arachidonic acid to prostaglandins (PGE), leading to increased osteogenic gene expression (Choudhary et al., 2003). The osteogenic effect of Sr has been shown to depend on PGE2, since the inhibition of COX-2 reduces Sr-induced production of PGE2 and the subsequent stimulation of ALP gene expression in MSCs (Choudhary et al., 2007). This occurs *via* a CaSR-dependent and independent pathway: COX-2 inhibition reduces Sr mediated effects in both CaSR−/− and CaSR+/+ cells (Fromigué et al., 2009). Another important pathway effected by Sr is the Akt pathway, which promotes survival by inhibiting apoptosis in osteoblasts (Rybchyn et al., 2011). Sr has been shown to activate this pro-survival pathway in both CaSR−/− and CaSR+/+ cells, pointing to the presence of another receptor partially mediating this effect.

**Fig. 1:**
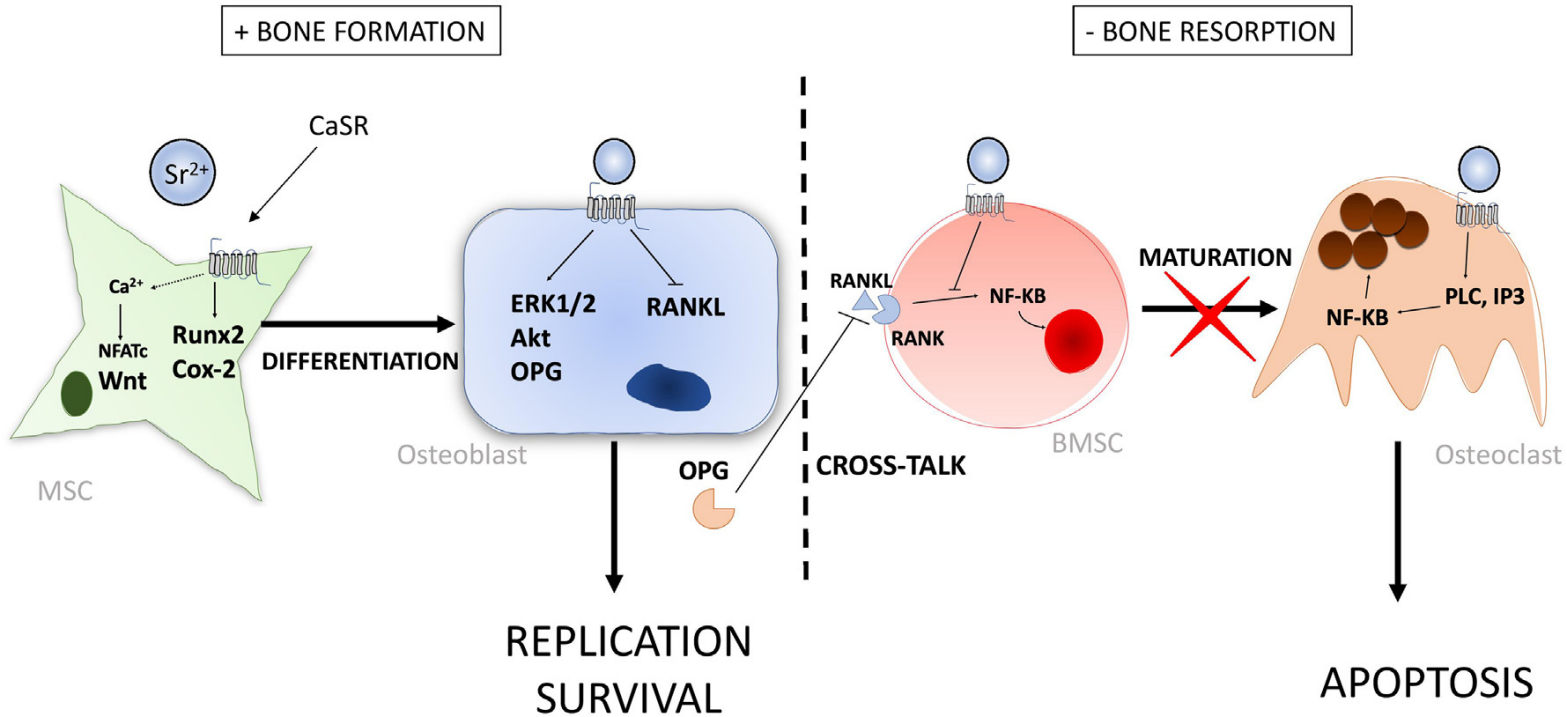
Cellular CaSR-dependent mechanisms for Sr’s action on osteoblasts and osteoclasts from *in vitro* findings. (Hurtel-Lemaire et al., 2009; Rybchyn et al., 2011; Peng et al., 2009; Fromigué et al., 2009; Peng et al., 2011b; Caverzasio, 2008).

Sr has been shown to mediate calcineurin (Cn)/ nuclear factor of activated Tc (NFATc) signalling in osteoblasts (Asagiri et al., 2005). NFATc is a transcription factor that resides in the cytoplasm of osteoblast cells (Hogan, 2003; Crabtree and Olson, 2002) and calcineurin (Cn) is a phosphatase that becomes activated in response to rising intracellular Ca^2+^. Upon activation by Ca^2+^, Cn dephosphorylates NFATc inducing its translocation into the nucleus, where it binds to target genes such as Runx2 and ALP (Koga et al., 2005; Yeo et al., 2007). SrRan treatment of pre-osteoblast cells *in vitro* has been shown to induce the nuclear translocation of NFATc (Fromigué et al., 2010) and Cn inhibitors have been shown to completely inhibit the increased expression of Runx2 and ALP observed after Sr treatment. Interestingly, Ca^2+^-treatment also activates this pathway. Since CaSR stimulation causes an influx of intracellular Ca^2+^, it is very plausible that Cn-NFATc signalling is indirectly activated by CaSR stimulation and runs parallel to ERK/MAPK signalling. Cn-NFATc has been shown to promote the expression of Wnt genes in response to Sr treatment in osteoblasts. Wnt signalling is a major pathway regulating the osteogenic differentiation of MSCs, and disruption of signalling molecules involved in this pathway is closely linked to pathologic bone conditions (Kim et al., 2013). SrRan induces the expression of both canonical and non-canonical Wnt proteins, and this effect is abolished with use of the Cn-inhibitors (Fromigué et al., 2010). Additionally, Sr treatment induces the translocation of ß -catenin into the nucleus (canonical), and DKK1 (an inhibitor of the non-canonical pathway) reduces the expression of osteogenic genes such as ALP and Runx2. Nuclear translocation of ß -catenin usually occurs due to extracellular bindings of Wnt proteins; however, Sr-induced translocation has been shown to be mediated in part by Akt activation, downstream of CaSR (Rybchyn et al., 2011). This occurs because Akt is able to phosphorylate ß -catenin, increasing its transcriptional activity (Fang et al., 2007). The cellular CaSR-dependent mechanisms for Sr’s effect on osteoblast cells is summarized and shown in Fig. 1.

#### 2.2.3 Mineralization

Differential effects on mineralization have been observed in osteoblast cultures in response to Sr treatment. In human osteoblasts, Sr (0.1- 2 mM) has been shown to significantly increase mineralization after 14 days, compared to controls (Rybchyn et al., 2011). Similar effects have been found in mouse osteoblasts exposed to 0.1-1 mM Sr (Bonnelye et al., 2008). In MC3T3-E1 cells, Sr doses of 0.1-1 mM have been shown to have no effect on mineralized nodule formation (Barbara et al., 2004). Interestingly, Sr at doses of 0.1-1 mM have been shown to significantly inhibit mineralization in primary rat osteoblasts, while maintaining collagenous nodule formation (Wornham et al., 2014; Verberckmoes et al., 2003). It has been suggested that this effect is mainly due to a physiochemical mechanism, whereby the incorporation of Sr into HA slows down crystal growth and increases the solubility of the mineral, which explains why collagen production was normal. This has also been observed with synthetic Sr-incorporated HAs that have reduced crystal size and increased solubility compared to controls (Verberckmoes et al., 2004). While these findings contrast with the majority of *in vitro* reports, it's important to consider how different culture conditions may alter results, which is a huge limitation to *in vitro* experiments. These findings, however, do agree with some *in vivo* studies in chronic renal failure rats which developed osteomalacia after Sr administration due to high accumulation of Sr in bone (Oste et al., 2005). Defective mineralization has not been observed in humans, however, some pre-clinical trials (using high Sr doses) and novel clinical trials have reported decreased bone formation in response to Sr administration, which is discussed in Section 3.

### 2.3 Effects of Sr on osteoclast cells

Osteoclast precursors and mature osteoclasts express the CaSR on their cell membrane, and therefore, we will first briefly discuss the effect that Ca exerts on osteoclasts and relate this to Sr’s effect. Osteoclasts are exposed to high Ca concentrations within resorption pits (Silver, 1988), and therefore direct Ca sensing may play a role in controlling their function. Indeed, it has been shown experimentally that the CaSR is able to control osteoclast maturation and apoptosis in response to extracellular Ca (Mentaverri et al., 2006; Lorget et al., 2000). The differentiation of bone marrow stromal cells (BMSCs) into mature osteoclasts is dependent on nuclear factor-kappa B (NF-κB) signalling, which is activated in response to receptor activator of nuclear factor kappa B ligand (RANKL) (Boyce et al., 2015). Osteoblasts play an important role in this process because they secrete soluble RANKL, which binds the corresponding receptor RANK on precursor osteoclasts, leading to their differentiation and maturation (Boyce and Xing, 2008). Interestingly, Ca stimulation of CaSR also promotes the nuclear translocation of NF-kB and subsequent differentiation of BMSCs into mature osteoclasts, which involves the activation of phospholipase C (Lorget et al., 2000). It is therefore not surprising that BMSCs isolated from CaSR knockout mice are unable to differentiate into mature osteoclasts. Conversely, high Ca has been shown to induce apoptosis in mature rabbit osteoclasts mediated by CaSR, which leads to decreased resorptive activity (Zaidi et al., 1991). Here we will discuss how Sr stimulation differs from Ca stimulation in terms of osteoclast function.

#### 2.3.1 Formation and maturation

Sr treatment has been shown to dose-dependently decrease osteoclastogenesis and therefore reduce the number of mature osteoclasts *in vitro* (Bonnelye et al., 2008; Baron and Tsouderos, 2002), which is directly related to a reduction in the formation of resorption pits. This effect on differentiation is contrary to Ca stimulation at early stages. The use of a dominant-negative CaSR construct has been shown to partially reduce Sr-mediated effects in osteoclasts, strengthening the hypothesis that these cellular effects are mediated by the CaSR (Caudrillier et al., 2010). Reduced formation of osteoclast cells was accompanied by the inhibition of RANKL-mediated nuclear translocation of NF-kB. However, it should also be noted that inhibited osteoclast formation is only partially responsible for reduced resorbing activity. It has been shown that Sr alters the actin cytoskeleton of osteoclasts at the sealing zone, which disrupts ruffled border formation and reduces the surface area available for proton exchange (Bonnelye et al., 2008; Takahashi et al., 2003). This disruption has been shown to significantly inhibit resorbing activity, irrespective of changes in osteoclast number (Takahashi et al., 2003). While CASR activation (with the use of calcimimetics) has been shown to induce cytoskeletal changes in epithelial cells (Abdulnour-Nakhoul et al., 2015), the mechanism for this effect in osteoclasts has not yet been investigated.

#### 2.3.2 Cross-talk

Sr has been shown to affect the cross-talk between osteoblasts and osteoclasts, which plays an important role in controlling osteoclast differentiation. Osteoblasts also secrete osteoprotegrin (OPG), which acts as a decoy receptor by binding to RANKL, promoting osteoclast apoptosis (Boyce et al., 2015). OPG has been found to play a central role in Sr’s effect on bone, since OPG knockout mice do not have the reduced bone resorption and subsequent increase in trabecular volume that is seen in wild-type mice after Sr treatment (Peng et al., 2011a). Sr can increase osteoblastic OPG mRNA expression and reduce RANKL expression *in vitro* (Peng et al., 2011b), and knockdown of CaSR results in the suppression of this effect, as well as the subsequent decrease in osteoclastic cell replication.

#### 2.3.3 Apoptosis

Sr has been shown to dose-dependently stimulate apoptosis in mature osteoclasts, similarly to Ca stimulation. For Ca, this is mediated by the activation of PLC and the production of inositol phosphate (IP), which leads to the nuclear translocation of NF-kB. The intracellular signalling cascade differs from that of Ca for mature cells in that Sr-stimulated apoptosis is dependent on PKC activation and independent of IP (Hurtel-Lemaire et al., 2009). The combination of Sr and Ca is able to stimulate apoptosis to a greater extent than the individual action of either ion, suggesting they act on different cellular signalling cascades to potentiate the response. The effect of Sr on osteoclast cells is summarized and shown in Fig. 1.

### 2.4 Other cation-sensing receptors

While the CaSR has been found to be expressed in bone cells, it is unclear whether this receptor plays a primary role in cation sensing. Primary osteoblast cells isolated from CaSR-deficient mice are still able to respond to Ca and Sr (Pi and Quarles, 2004). Additionally, while osteoclast precursors and mature osteoclasts cells have been found to express CaSR *in vitro*, transcripts were rarely present in osteoclasts *in vivo*, assessed by *in situ* hybridization (Chang et al., 1999). While the signal transduction cascades for CaSR in response to Sr and Ca have been extensively studied in various cell types, multiple reports have not been able to detect CaSR in human cell lines (Pi et al., 1999). Another extracellular cation sensing receptor, GPRC6A, has been identified that is expressed by bone cells and can sense extracellular cations such as Ca and Sr (Pi et al., 2005). Osteoblasts isolated from GPRC6A−/− null mice have reduced expression of osteogenic markers, and null mice exhibit osteopenia with reduced BMD and bone mineralization (Pi et al., 2009). It should be noted that in this report, the mutation was not specific to osteoblast cells and deficient mice additionally exhibited hormone abnormalities making the true role that GPRC6A plays in bone remodelling unclear. Research on the specific effect that this receptor has in bone cells is still needed, with some conflicting reports which show that GPRCC6A−/− mice exhibit no bone phenotype under physiological conditions (Wellendorph et al., 2009). It is quite possible that many more cation sensing receptors exist that mediate Sr’s effect on bone cells.

There is evidence that the fibroblast growth factor receptor (FGFR) can respond to Sr and Ca, mediating osteoblast growth *via* a CaSR-independent mechanism. FGF is produced by osteoblasts and binds FGFR on osteoblasts in a paracrine/autocrine fashion to promote cell growth (Takei et al., 2015), and selective inhibitors of FGFR are able to attenuate Sr-mediated cell growth (Caverzasio and Thouverey, 2011). Interestingly, Sr administration has no effect on the levels of FGF produced by osteoblast cells, suggesting that Sr directly interacts with FGFR. Sr was able to activate downstream signalling molecules of FGFR such as ERK and fibroblast receptor substrate 2 (FRS2) as rapidly as FGF. FGF/FGFR signalling is critical for skeletal development, which is apparent in mice deficient for FGF2 that develop abnormal trabecular microarchitecture and decreased bone mass (Montero et al., 2000). This may explain why CaSR inhibitors were only able to decrease Sr-mediated effects, instead of inhibiting them completely.

## 3 Effects of Sr *in vivo*

### 3.1 Pre-clinical trials

*In vitro* experimental studies have provided ample evidence for Sr’s anabolic and anti-resorptive effects, which strengthens support for the dual-acting mechanism. Pre-clinical trials also present experimental evidence that supports Sr’s uncoupling effect on bone remodelling. Sr administration (316-350 mg/kg/day SrCl_2_) has been found to increase bone mineral density, bone mass and osteoid volume in normal rodents, without disturbing bone mineralization (Marie et al., 1985; Marie and Hott, 1986; Grynpas and Marie, 1990). One of these studies also found the number of active osteoclasts on the endosteal surface of rat vertebra to be decreased in the Sr-treated group compared to controls, evaluated by bone histomorphometry (Marie and Hott, 1986). A later study found that a low dose of Sr (168 mg/kg/day) increased the number of bone-forming sites in the vertebra of intact rats, significantly increasing trabecular bone volume compared to controls (Grynpas et al., 1996). In terms of toxicity, Sr has been shown to interfere with normal Ca metabolism at doses higher than 510 mg/kg/day in normal rats fed low Ca diets, resulting in hypocalcaemia, altered Ca intestinal absorption, decreased serum 1,25-D and defective bone mineralization (Morohashi et al., 1994; Marie et al., 1985; Grynpas and Marie, 1990). Doses over 510 mg/kg/day have also been reported to induce a global inhibition of bone remodelling (both formation and resorption) leading to increased trabecular bone volume. During bone normal development, remodelling in trabecular bone leads to the removal of mineral and the formation of a marrow cavity, whereas growing rats fed high doses of Sr (above 510 mg/kg/day) did not fully form marrow cavities which resulted in increased bone volume (Morohashi et al., 1994; Setiawati and Rahardjo, 2019). Despite the toxic effects at high doses, later pre-clinical trials found significant increases in serum bone-alkaline phosphatase (b-ALP) (+53% *p* < 0.001), a marker of bone formation, in rats fed 625 and 900 mg/kg/day of SrRan for 2 years, accompanied by increased bone volume and bone strength (maximal load) (Ammann et al., 2004; Ammann et al., 2007). Using micro-computed tomography (μ-CT) and nano-indentation techniques integrated with finite-elemental (FE) analysis, studies have shown that SrRan (900 mg/kg/day) improves trabecular thickness and cortical thickness alongside modulus, hardness and dissipated energy *versus* controls (Ammann et al., 2007; Boyd et al., 2011). In ovariectomized (OVX) animals, Sr administration has been shown to reduce the increased bone remodelling associated with estrogen deficiency. Sr administration (50 mg/kg/day) decreased both bone formation and bone resorption in OVX rats, however, this did not produce any effect on bone volume (Morohashi et al., 1995). Additionally, Sr doses of 77, 154 and 308 mg/kg/day prevented bone loss in OVX rats by a similar degree as estrogen administration (Marie et al., 1993). Later studies found that SrRan treatment (625 mg/kg/day) completely restored the intrinsic tissue quality of bone from OVX rats back to SHAM levels, evidenced by significantly increased maximal load and energy to fracture compared to OVX controls (Bain et al., 2009). Additionally, some aspects of bone microarchitecture were restored, such as trabecular volume, trabecular number and trabecular spacing.

The majority of pre-clinical trials discussed above fed rats relatively low Ca diets (~0.5–0.76%) (Table 1). Sr administration taken together with a low Ca diet has been shown to enhance the effect of Sr in bone, due to higher accumulation in bone mineral and increased serum Sr levels (Fuchs et al., 2008). Since the effect of Sr may be amplified in these studies, the results are difficult to extrapolate to clinical trials since osteoporotic patients receiving SrRan were required to take calcium supplements. Additionally, the Sr doses used in pre-clinical trials have been criticized since increases in bone strength in rats were only observed for doses over 625 mg/kg/day, which is above the toxic dose found in previous studies (Morohashi et al., 1994; Marie et al., 1985; Grynpas and Marie, 1990). The therapeutic dose later administered in clinical trials is 2 g/day (~25 mg/kg/day), which has been found to produce serum Sr levels in rats that align with those found in humans. Indeed, the 900 mg/kg/day dose in rats leads to a serum Sr level which is double the therapeutic level for humans (Boyd et al., 2011). More recent studies have shown no effect on bone formation or bone volume in OVX rats when they are administered 25 mg/kg/day and a normal Ca diet (1.19%) (Fuchs et al., 2008).

**Table 1:** Histomorphometric effects of Sr for different doses and calcium diets. At pharmacological doses and normal/supplemented calcium diets, Sr has not been shown to have a significant anabolic effect.

Article	Subject	Form of Sr	Sr dose (mg/kg/day)	Duration of treatment	Calcium diet	Effects
Marie et al. (1985)	WT Rat	SrCl_2_	316, 633.7	9 weeks	0.50%	↑ trabecular bone density and calcified bone growth (316 dose) ↓ serum calcium and 1,25-D, induced defective mineralization (633.7 dose)
Morohashi et al. (1994)	WT Rat	SrCO_3_	510	27 days	0.50%	↑ trabecular bone volume ↓ bone formation, bone resorption, calcium content in bone (−20%) and serum calcium
Marie and Hott (1986)	WT Rat	SrCl_2_	350	29 days	0.50%	No significant effect on bone volume or bone mineral content ↑ osteoid surface by 10% ↓ number of active osteoclasts by 11%
Grynpas et al. (1996)	WT Rat	omitted	168	8 weeks	0.50%	↑ mineral bone volume by 17% ↑ number of bone-forming sites by 70%, with no adverse effect on mineralization
Grynpas and Marie (1990)	WT rat	SrCl_2_	316, 633.7	8 weeks	0.50%	↑ osteoid volume and bone mass with no effect on bone volume ↑ bone volume and parameters of bone formation, accompanied by defective bone mineralization
Ammann et al. (2004)	WT rat	SrRan	225, 450, 900	2 years	0.76%	↑ bone volume (625 and 900 doses) ↑maximal load (900 dose only)
Morohashi et al. (1995)	OVX Rat	SrCO_3_	50	2 weeks	0.50%	↓bone formation and bone resorption, but not completely back to sham levels. No change in bone volume in any group.
Marie et al. (1993)	OVX Rat	SrRan	77, 154, 308	2 months	0.60%	↑ bone volume by 30–36% ↓ osteoclast number/ surface no effect on osteoblast surface/number
Fuchs et al. (2008)	OVX Rat	SrRan	25, 150	90 days	0.1% or 1.19% (normal)	No effect on bone volume, bone formation observed for either dose or calcium diet ↑ serum Sr and Sr accumulation in bone for low calcium diet
Arlot et al. (2007)	Biopsies from PREVOS, STRATOS, SOTI and TROPOS	SrRan	25	1–5 years	Normal +500 mg/day	↑ BMD and ↓ new fractures for all clinical trials ↑osteoblast surface and osteoid thickness no effect on bone resorption was observed^a^
Chavassieux et al. (2014)	Post-menopausal osteoporotic women	Sr-Ran	25	6–12 months	Normal +1000 mg/day	↓ mineralizing surface/bone surface (MS/BS) no effect on resorption ↓ bone volume, trabecular thickness and trabecular number

↑ Increase ↓ Decrease

a Only 4 paired treatment biopsies and 1 paired placebo biopsy performed.

### 3.2 Clinical trials

#### 3.2.1 Bone strength

Phase 2 and 3 clinical trials for SrRan have shown that it significantly reduces fracture risk in post-menopausal women. Microarchitecture is an important aspect of bone strength and increased fracture risk in patients with osteoporosis is associated with trabecular thinning and increased cortical porosity (Osterhoff et al., 2016). Enhanced bone microarchitecture has been reported after SrRan treatment in postmenopausal women, possibly explaining reduced fracture risk. One study analyzed unpaired biopsies from SOTI, TROPOS and STRATOS clinical trials (*n* = 41) using μ-CT imaging to assess microstructure, and determined that SrRan treatment significantly increased cortical thickness (18%) and trabecular number (14%) compared to the placebo group (Arlot et al., 2007). In a more recent study, SrRan treatment was shown to increase cortical thickness (6.3%), trabecular number (3.6%) and decrease trabecular spacing (3.0%) from baseline, determined using high-resolution peripheral quantitative computed tomography (HR-pQCT) (Rizzoli et al., 2012). Compromised architecture during the pathogenesis of osteoporosis is associated with bone remodelling; loss of bone occurs since bone formation is not equal to bone resorption. However, bone strength also depends on the intrinsic material quality of bone, which has also been shown to be improved in response to Sr treatment. In postmenopausal women, Sr has been shown to increase failure load by 2.1% (*p* < 0.005) from baseline, determined using HR-pQCT and FE analysis. In this study, no change in predicted failure load or cortical thickness was observed for patients receiving alendronate treatment, a potent anti-resorption drug (Rizzoli et al., 2012).

#### 3.2.2 Bone-mineral density

Bone mineral density (BMD) measured by dual-energy X-ray absorptiometry (DXA) is a parameter used clinically to diagnose osteoporosis and determine the therapeutic effect of anti-osteoporotic treatments. A major finding of the SOTI, PREVOS, STRATOS and SOTI clinical trials was that SrRan treatment significantly increased BMD at the lumbar spine, hip and femoral neck (Reginster et al., 2002)–(Reginster et al., 2005). Additionally, increases in BMD are correlated with reduced fracture risk in patients treated with SrRan. However, BMD measurements have been disputed in recent years (Blake et al., 2009; Blake and Fogelman, 2013) since they may not be the result of an increase in bone mineral content, but rather the result of Sr’s ability to replace Ca in bone. Sr’s larger atomic number (Z = 38) when compared to Ca (Z = 20) causes attenuation of the X-ray leading to an overestimation of BMD that does not reflect a true change in the amount of bone tissue (Blake and Fogelman, 2006). If the Sr content in bone is 1% (1% of Ca has been replaced by Sr on a molar basis), then the BMD measured by DXA will be overestimated by 10%, which is referred to as the Pors Neilsen factor (Nielsen et al., 1999). This was determined by mixing different amounts of Sr with synthetic HA and measuring the effects using DXA. It should be noted that this conversion does change slightly depending on the different DXA machines used due to variations in photon energy (Nielsen et al., 1999).

It is possible that most of the increases in BMD seen in clinical trials are the result of X-ray attenuation, since bone biopsy specimens from patients receiving Sr for three years have approximately 1.6% Sr content (Boivin et al., 2010) and the SOTI study reported a 14% increase in spine BMD and a 10% increase in hip BMD (Meunier et al., 2004). However, the Pors Neilson conversion factor is just one part of the equation, to estimate the BMD overestimation it is also required to know the ratio (R) between the bone Sr content (BSC) at the DXA site (spine) and the BSC at the iliac crest (biopsy site). One study sought to estimate the BMD error using the DXA data from the four clinical trials and the BSC found in the iliac crest of monkeys fed proportional doses of Sr from a previous study (Blake and Fogelman, 2006). They found that Sr incorporation could account for 75–100% of BMD measurements, however the assumption of R provides obvious limitations. Nevertheless, there is a strong relationship between reduced fracture risk and increased BMD in patients following Sr administration (Blake and Fogelman, 2006), which supports a purely physiochemical mechanism to explain increases in bone strength independent of changes in bone formation/resorption. It should also be noted that some studies reported here used HR-pQCT to assess changes in bone microstructure in response to SrRan treatment (Rizzoli et al., 2012; Rizzoli et al., 2010). Positive changes in microarchitecture should also be interpreted with caution due to the unknown effect of X-ray attenuation caused by Sr’s larger atomic nucleus. However, no present studies have assessed the degree by which Sr produces artifacts in HR-pQCT measurements.

#### 3.2.3 Parameters of bone formation and resorption

Phase 2 and 3 clinical trials for SrRan have shown that it reduces fracture risk, increases BMD and alters some biochemical markers of bone remodelling in favour of the ‘dual acting mechanism’. However, these initial clinical trials were unable to support the claim that Sr has any anabolic effect due to a severe lack of paired biopsy specimens (Arlot et al., 2007). The mechanism of action of an anti-osteoporosis drug cannot be conclusively determined without histomorphometric analysis of paired transiliac bone biopsies, and unfortunately, only 5 patients had baseline data collected (*n* = 1 control and *n* = 4 Sr treatment). As a result, histomorphometric analysis was unable to show that SrRan could stimulate bone formation significantly after 1–5 years of treatment, compared to placebo. There was evidence that Sr treatment improved some parameters of bone formation such as cortical mineral apposition rate and cortical osteoblast surface, however, these changes were not accompanied by an increase in bone volume.

Clinical trials have found that SrRan treatment positively influences some serum and urinary biochemical markers of bone remodelling. However, while clinical trials did not have a shortage of biochemical data, these findings have been disputed due to inconsistencies and small magnitude changes compared to other anti-osteoporotic drugs (Blake et al., 2009). The PREVOS trial found a 41% (*p* = 0.048) increase in b-ALP after 4 months for the group receiving 1 g/day (Reginster et al., 2002). This result has been questioned in part because later studies were unable to replicate it with the approved pharmacological dose of 2 g/day, and because b-ALP also increased simultaneously in the placebo group during the 1-year study. In contrast, the SOTI and STRATOS trials found an 8–11% increase in b-ALP after 3 months when administering the appropriate dose of 2 g/day (Meunier et al., 2002; Meunier et al., 2004). To put this into perspective, the anabolic agent PTH(1–84) has been shown to increase b-ALP by 73% and 129% after 12 weeks and 24 weeks respectively (Quesada-Gómez et al., 2011). In addition, the PREVOS trial found that OC, another marker of bone formation, increased in both the treatment and placebo group, making the results insignificant. The STRATOS trial similarly found no significant change in OC, or procollagen. It seems that the pharmacological dose was not sufficient to induce large magnitude changes in positive markers of bone formation, which has been criticized by two publications (Blake et al., 2009; Blake and Fogelman, 2013).

Markers of bone resorption measured in clinical trials include urinary cross-linked C-terminal telopeptide of type 1 collagen (CTX), serum CTX and urinary N-telopeptides of type 1 collagen (NTX). The PREVOS trials found no significant changes in urinary CTX between SrRan treated groups and the control (Reginster et al., 2002), while the SOTI study found a 12.2% (*p* < 0.001) decrease in serum CTX in the treatment group after 3 months (Meunier et al., 2004). The STRATOS trial found a 20.2% (*p* = 0.004) decrease in urinary NTX in the treatment group after 1 year (Meunier et al., 2002). In comparison, one study found that alendronate treatment, a potent anti-resorptive agent, decreased serum CTX levels by 30.4% and serum NTX levels by 43.5% after 6 months, continuing to decrease NTX to 67.3% after 2.5 years (p < 0.001) (Greenspan et al., 2000). The SrRan clinical trials found serum/urinary markers to plateau after 3/6 months, contrary to alendronate treatment. From these results, it appears that Sr does have an inhibitory effect on bone resorption, but to a lesser degree than more potent agents.

Several recent studies have compared Sr treatment directly to other anti-osteoporotic drugs using a large sample size of paired biopsy data, with the aim of conclusively determining Sr’s mechanism of action (Quesada-Gómez et al., 2011; Chavassieux et al., 2014; Recker et al., 2009). In a study by Chavassieux et al. (Chavassieux et al., 2014) 256 patients received SrRan treatment and 131 patients received alendronate treatment. All patients underwent a biopsy at baseline, as well as at 6 or 12 months, and out of the 387 paired biopsies, 286 were used for analysis. As expected, alendronate significantly decreased both parameters of resorption and formation, resulting in a lower rate of bone turnover that maintained bone mass and microarchitecture. Surprisingly, SrRan was seen to significantly decrease some parameters of bone formation such a mineralizing surface/bone surface (MS/BS), but to a lesser extent than alendronate. SrRan treatment had no effect on resorption, which led to a decrease in bone volume (*p* = 0.011), trabecular thickness (*p* = 0.024) and trabecular number (p < 0.001) at 12 months. Interestingly, 67.6% of the patients in the SrRan group experienced a decrease in MS/BS, while 32.4% experienced an increase in MS/BS. The experimenters found that this subgroup of patients that experienced an increased in MS/BS at 12 months had a low rate of remodelling at baseline. This study was the first to use a large number of paired biopsies to evaluate Sr’s effect, and these findings do not support any anabolic action, and instead, the authors propose that Sr’s mechanism of action is purely physiochemical.

Recker *et al (*Recker et al., 2009*)* compared the effects of SrRan to the anabolic drug teriparatide (human recombinant PTH(1–34)) and assessed biochemical markers such as serum amino-terminal propeptide of type I collagen (PINP), a marker of bone formation as well as b-ALP and CTX. Consistent with previous reports, teriparatide increased all of these markers significantly (PINP: 131%, b-ALP: 32.4% and B-CTX: 111%). This effect is consistent with previous reports and is referred to the ‘anabolic window’: markers of resorption increase after markers of formation since the two processes are coupled, leading to a net anabolic effect. Conversely, individuals receiving SrRan exhibited a drop in all of these markers over the course of the study, which was significant for the markers PINP and CTX. This study also utilized paired biopsy data (*n* = 29 for teriparatide, *n* = 22 for SrRan) and found that the dynamic parameters of bone formation were consistently higher in the teriparatide group but were statistically insignificant. The authors noted that the mean value for MS/BS for teriparatide was lower than previous studies, which may be due to the omittance of endocortical envelopes from their evaluation fields or the result of variations in study population. This study therefore does not present any evidence for an anabolic effect in response to Sr treatment and agrees with Chavassieux et al. that Sr decreases markers of bone formation (Chavassieux et al., 2014).

Quesada-Gómez et al. (Quesada-Gómez et al., 2011) compared the effect of SrRan to PTH(1–84) over the course of 24 weeks in post-menopausal women with osteoporosis (SrRan *n* = 40, PTH *n* = 41). They found that SrRan treated patients had no significant changes in PINP, b-ALP or CTX from baseline. There was a modest increase in b-ALP at 4 months, decrease in PINP throughout the course of the study and a decrease in CTX throughout (−10.7%). These findings are similar to those seen in the phase 3 clinical trials for SrRan. Similarly to the study from Recker *et al* and previous reports, PTH(1–84) treatment results in a large and sustained increase in the markers of bone formation (+446.1% in PINP and + 129% in b-ALP at 24 weeks) and a delayed increase in markers of resorption (+112% in CTX), indicating active turnover. These relatively new clinical trials question whether SrRan has an anabolic effect on bone, with some studies indicating that it may have a weak anti-remodelling effect. From the three studies discussed here, it appears that Sr administration results in a global inhibition of bone remodelling, which may be beneficial in the treatment of a disease characterized by high turnover. As previously mentioned, bisphosphates decrease both parameters of bone formation and bone resorption to maintain or increase BMD in the treatment of osteoporosis. Perhaps the subtle changes in bone formation/resorption are sufficient to slow down the rapid bone loss seen in osteoporosis, or conversely, the incorporation of Sr into bone may simply increase bone strength due to physiochemical mechanisms alone.

## 4 Physiochemical mechanisms

### 4.1 Strontium incorporation in bone

Sr has been shown to dose-dependently incorporate directly into bone, both in animal studies (Morohashi et al., 1994; Morohashi et al., 1993; Marie et al., 1985; Marie and Hott, 1986) and clinical trials (Boivin et al., 2010). It has also been found to incorporate into the mineral formed during *in vitro* culturing of osteoblast cells (Verberckmoes et al., 2003). The substitution of Sr for Ca in HA leads to the expansion of the crystal lattice, due to Sr’s larger ionic radius. It has been found experimentally that lattice parameters a and c increase linearly with increasing Sr in synthetic HA's (Bigi et al., 2007). Since these parameters increase linearly, they have been used to estimate the degree by which Sr replaces Ca in the HA crystal lattice, both in animals and humans (Querido et al., 2016). In terms of crystal morphology, a reduction in crystal size and crystallinity has been observed in synthetic HA's with 15% Sr (relative to Ca), 24% Sr and 64% Sr (Aina et al., 2013; Li et al., 2007). Decreased crystal size has also been observed in the bones of rats receiving 633 mg/kg/day of Sr, measured using X-Ray diffraction (XRD) (Grynpas and Marie, 1990). However, this effect was not observed for the lower dose of 316 mg/kg/day. Moreover, no change to crystal size or crystallinity were observed in bone biopsies taken from post-menopausal women administered 2 g/day of SrRan, assessed using XRD (Li et al., 2009).

**Fig. 2:**
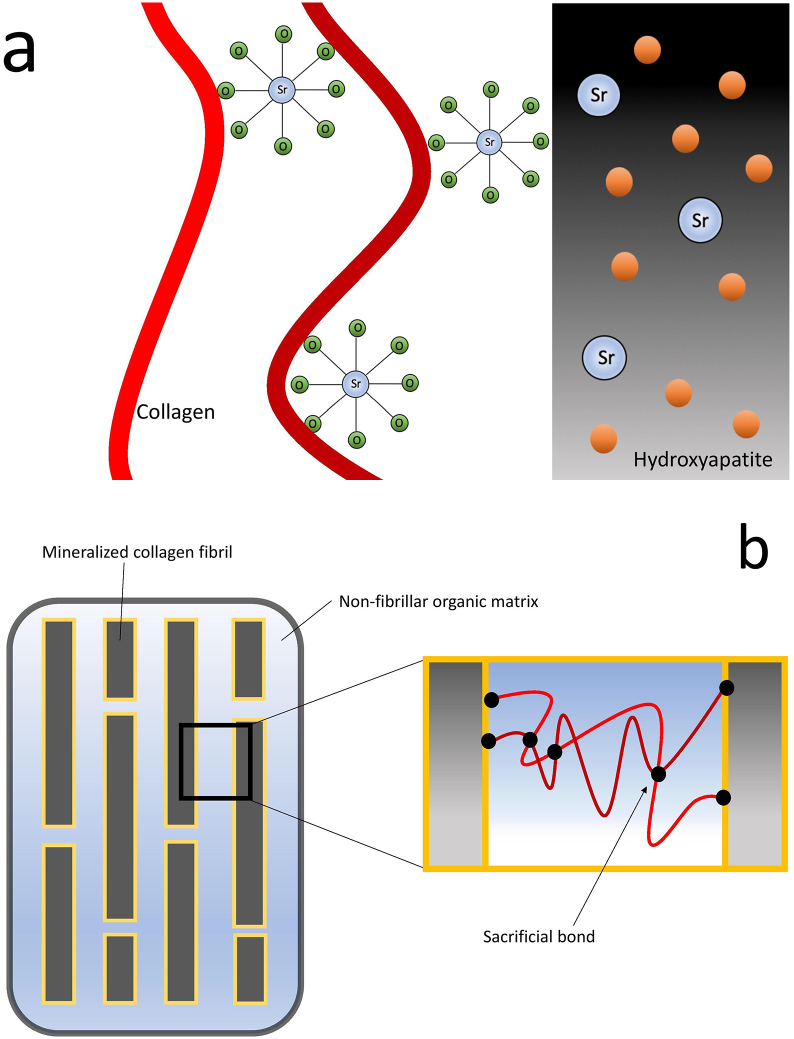
a) Localization of Sr in bone. Adapted from Frankær et al.(Frankær et al., 2014) b) Nanostructured organization of bone as well as the location of sacrificial bonds. Adapted from Gao et al. (2004).

In animals that have not been treated with Sr, the element is uniformly distributed in bone, whereas treated animals have heterogeneous distribution of Sr, with a preference for newly formed bone rather than old bone. Sr has a preference for young bone since it is less mineralized, allowing Sr to readily exchange with loosely bound ions contained in the layer of water which surrounds HA crystals (Cazalbou et al., 2005)–(Cazalbou et al., 2004). As the crystal matures and the hydrated layer is replaced by mineral, Sr irreversibly incorporates into the mineral lattice (Querido et al., 2016). This explains why, in an experiment with monkeys, Sr content in bone rapidly decreased when treatment was terminated, since the majority of Sr was not irreversibly incorporated into HA crystals and instead was present outside the crystals (Farlay et al., 2005). In an experiment where dogs were fed Sr-malonate, x-ray absorption spectroscopy analysis found that only 35%–45% of the Sr incorporated into bone was present in the HA crystal lattice, the remainder of Sr was either adsorbed onto the crystals surface, bound to the collagen matrix, or present in bone fluids (Fig. 2A) (Frankær et al., 2014). Therefore, changes in intrinsic tissue quality following Sr-treatment are more likely related to its interaction with collagen, water and the hydrated layer surrounding crystals, rather than its ability to incorporate directly into HA.

### 4.2 Strontium and sacrificial bonds

Sr-mediated sacrificial bond formation within the organic matrix is one proposed mechanism to explain changes in intrinsic tissue quality following Sr-treatment. Bone is a nanocomposite material composed of a mineral phase which is embedded in an organic matrix (Fig. 2B). The mineral portion consists of mineralized fibrillar collagen with non-stoichiometric nanostructured HA, while the organic matrix consists mainly of a non-fibrillar type 1 collagen matrix, along with water and non-collagenous proteins (Stock, 2015). Weak bonds in the organic matrix, termed sacrificial bonds, are able to break and reform, allowing organic polymers to dissipate large amounts of energy before breaking by revealing hidden length (Fantner et al., 2006). This toughening mechanism has also been shown to prevent crack formation in whole bone tissue by resisting the separation of mineralized fibrils (Fantner et al., 2005). Sacrificial bonds are formed and reformed *via* electrostatic interactions between negatively charged groups within a protein molecule or between different protein molecules, leading to inter and intra-molecular cross-linking (Fig. 2B). Additionally, these interactions may also form between non-fibrillar proteins and the mineral phase of bone. Current experimental evidence has shown that divalent ions such as Ca^2+^ mediate sacrificial bond formation, since mineralized fibrils are able to increase their energy dissipation over time in a Ca^2+^ buffer, but not in a Na^+^/K^+^ buffer (Fantner et al., 2005). It is therefore not surprising that the basis for Sr-induced changes in bone material quality are related to its similarity to *Ca.* Divalent ions such as Sr^2+^ and Ca^2+^ are able to form ionic bridges between polar groups of the organic matrix (unlike monovalent ions), reversibly linking protein modules together. These polar groups are possibly carboxylate groups on collagen and proteoglycans in the non-fibrillar matrix (Thompson et al., 2001).

However, when whole bones are soaked in a CaCl_2_ solution they do not have significantly greater yield strength or toughness when subjected to macroscopic mechanical testing (Thompson et al., 2001)–(Cattani-Lorente et al., 2013). It appears that the Ca^2+^ ions are not able to adequately penetrate bone to result in a significant effect, and only dissipate more energy than controls (bones soaked in sodium buffer) when non-destructively indented by 50 nm (Thompson et al., 2001). Bone samples soaked in SrCl_2_ do, however, have significantly increased material level properties such as elastic modulus (+19%), hardness (+42%) and working energy/toughness (+9%), assessed by nanoindentation (Cattani-Lorente et al., 2013). CaCl_2_ treatment similarly increased stiffness, but reduced hardness and had no effect on toughness, in cortical or trabecular bone. Cattani-Lorente et al. speculated that Sr^2+^ ions were able to diffuse into deeper locations within bones to produce these effects, since the (Sr + Ca)/P ratio in SrCl_2_ soaked bones is higher than the Ca/P ratio in CaCl_2_ soaked bones. Essentially, it appears that Sr ions do not only replace Ca in bone (as is expected) but they also occupy locations where Ca was previously absent, and are therefore able to form more sacrificial bonds between the matrix and mineral or between groups in the organic matrix (Cattani-Lorente et al., 2013). Additionally, Sr^2+^ treatment has a greater effect on bone taken from ovariectomized (OVX) rats, which had the largest observed changes in material properties, which may be due to a reduced degree of mineralization as a result of estrogen deficiency (Cattani-Lorente et al., 2013). However, experiments like these present obvious limitations, such as using ion concentrations that would normally not be found in the bone microenvironment.

Additionally, in an aqueous solution, Sr has been found to coordinate with 8 oxygen atoms at a distance of 2.57 Å, which has been determined using extended X-ray absorption fine structure (EXAFS) (Frankær et al., 2014; Seward et al., 1999). Frankær et al. speculated that Sr^2+^ would have a higher possibility of being involved in sacrificial bonds (either between collagen molecules or between collagen and mineral) since it has a larger ionic radius than Ca^2+^ (1.26 Å *vs* 1.12 Å for 8 coordinated oxygens). Therefore, Sr has the ability to form these electrostatic interactions over a greater distance, reaching further than Ca and increasing the possibility that a Sr^2+^ ion is involved in a sacrificial bond (Frankær et al., 2014).

### 4.3 Bound water

Another important and often overlooked constituent of bone is water, and there is a growing body of evidence that suggests that water bound to collagen and mineral is an essential contributor to bone's material properties, and may therefore contribute to Sr’s effect on bone strength (Granke et al., 2015). In one study, Sr was only found to positively affect bone material quality assessed by *ex vivo* nanoindentation when rat bones were under physiological conditions. Under dry conditions there was no significant effect between the placebo and treatment group (Ammann et al., 2007). Similarly, in bone samples taken from osteoporotic women treated with SrRan, no effect on mechanical properties was observed in dry conditions (Roschger et al., 2010). Water makes up 15–25% of total bone volume and exists mainly in two states; 1) free water is present within microscopic pores; and 2) bound-water is associated with the collagen matrix as well as the bone mineral (Granke et al., 2015). Experimental evidence has shown that water that is more loosely bound to collagen, and therefore more accessible, is strongly correlated to toughness (R^2^ = 0.81, *p* < 0.001) and post-yield toughness (R^2^ = 0.65, p < 0.001), while water that is associated with hydroxyproline (tightly bound to collagen molecules) is associated with strength (R^2^ = 0.48, p < 0.001) (Unal and Akkus, 2015). Additionally, water bound to mineral crystal (in the hydrated layer) is positively correlated with strength (R^2^ = 0.46, p < 0.001) inversely related to stiffness (R^2^ = 0.78, p < 0.001). As stated previously, Sr is mainly found localized to these compartments in bone, and therefore has been proposed to interact with, and possibly stabilize the bound water in these locations (Cattani-Lorente et al., 2013). However, to date, no direct experimental data has supported this mechanism. Given that Sr treatment has been shown to increase stiffness, hardness and toughness of bones (*ex vivo*), it's unlikely that sacrificial bond formation alone dictates these effects. It has been determined that mineral-bound and collagen-bound water contribute to these mechanical properties significantly and given that Sr mainly localizes to these compartments in bone, this mechanism of action is a plausible one.

## 5 Strontium in biomaterials

Sr has been incorporated into a wide variety of bone-implant materials, following the development of SrRan for the treatment of osteoporosis and growing reports that it positively influences bone remodelling. Given these beneficial effects both *in vivo* and *in vitro*, it seems plausible that locally delivered Sr ions will enhance osteoinduction and osseointegration at the bone-implant interface, reduce bone resorption, and ultimately contribute to faster healing after implantation. Despite the controversies surrounding Sr, Sr-modified bone materials have been shown to perform better than their Sr-free counterparts (Xue et al., 2006)–(Henriques Lourenço et al., 2017; Baier, 2006). In this section, the effects of Sr-doped materials such as Ca phosphate bioceramics, silicate/phosphate-based glasses/cements, functionalized titanium implants and microsphere/hydrogel hybrid systems will be presented. Evaluating Sr-enriched biomaterials *in vitro* and *in vivo* allows the determination of the effects of local ion delivery on bone and whether this may have an alternate effect compared to systemic administration. However, it is difficult to separate the effects that surface contact, local pH changes and the release of other ions such as Ca^2+^, phosphorus (P^5+^) and silicon (Si^4+^) may have on bone cell function and bone healing. Indeed, understanding how Sr release interacts with these variables to enforce the bone-implant interface may help to further understand Sr’s mechanism of action.

### 5.1 *In vitro*

Sr-leaching biomaterials have been shown to have enhanced bioactivity, and promote osteoblast attachment, proliferation and differentiation *in vitro*, to a greater degree than Sr-free materials. Synthetic hydroxyapatites doped with Sr have been found to promote the growth of osteoblast precursor cells on their surface better than HA alone, and moreover, lead to the accelerated expression of ALP, OC and collagen type 1 in these cells (Xue et al., 2006; Capuccini et al., 2009). Similarly, calcium-silicate glasses (Sr-CaSi's) doped with Sr have been shown to enhance the differentiation of osteosarcoma cells cultured on their surface, and this effect is dose-dependent with Sr-incorporation (Gentleman et al., 2010). Additionally, these materials have been shown inhibit the growth of osteoclasts more potently than Sr-free HA's or CaSi's (Capuccini et al., 2009; Gentleman et al., 2010). These findings are in agreement with the majority of earlier *in vitro* studies which looked solely at the effect of Sr^2+^ on cells. While Sr-CaSR binding is one mechanism to explain these effects *in vitro*, multiple variables are at play in these experiments, which we will outline in this section.

#### 5.1.1 Bioactivity

**Fig. 3:**
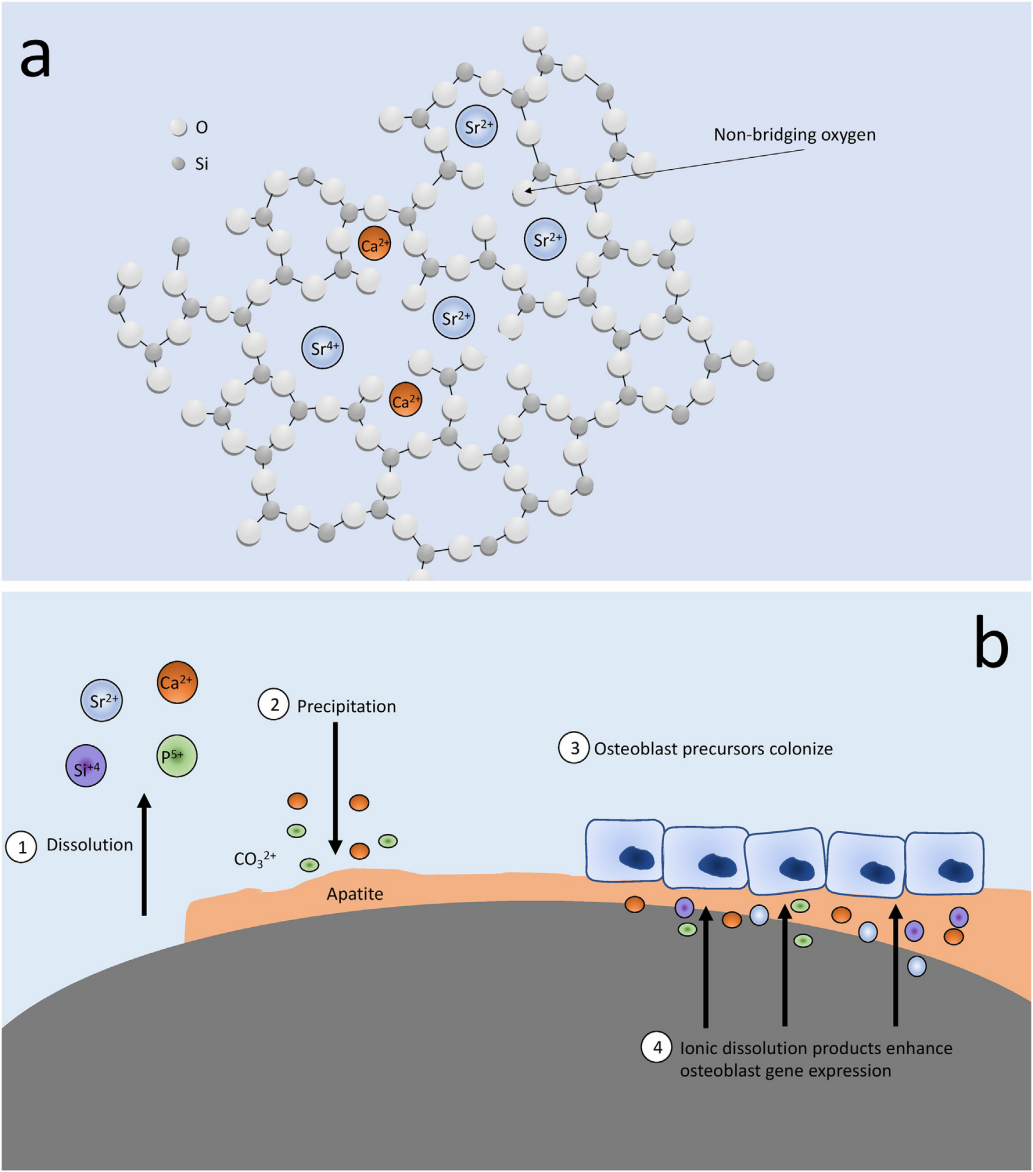
a) Structure of a Sr-CaSi glass, where the larger ionic radius of Sr^2+^ leads to a more expanded glass structure (Fredholm et al., 2012; Fredholm et al., 2010). b) Degradation/precipitation reaction of a bioactive Sr-CaSi glass, and how this favours osteoblast colonization, proliferation and gene expression.

Bioactivity is associated with enhanced cell attachment, proliferation and differentiation and in the case of bone-implant materials, it refers to their ability to spontaneously form a layer of HA on their surface in physiological conditions, allowing them to bond with host bone tissue (Jones, 2013)–(Hench et al., 2004). Sr-incorporation is known to increase the solubility of HA, which is related to bioactivity since the increased local concentration of Ca and P leads to increased precipitation of HA back on the surface of the material (Xue et al., 2006; Verberckmoes et al., 2004; Porter et al., 2003). After immersion in simulated body-fluid (SBF), HAs with 10 mol% Sr have a greater degree of spontaneous apatite precipitation on their surface than pure HA, which lends to enhanced bone-implant bonding *in vivo (*Capuccini et al., 2009*)*. Sr substitution for Ca in melt-derived CaSi glasses has similarly been shown to increase both the dissolution rate of the glass and subsequent apatite formation on the surface (Kargozar et al., 2019; Fredholm et al., 2012). This is due to the expanded and less rigidly bonded glass network, which is probably the result of the larger size of the Sr^2+^ ion, leading to more loosely bonded non-bridging oxygens (Fig. 3A) (Fredholm et al., 2010). Indeed, a more complete apatite layer on the surface of the material is surely a more suitable attachment site for osteoblasts (Fig. 3B), acting independently from the effects of Sr-release. It has been shown experimentally that surface characteristics can independently effect osteoblast production of ALP and OC (Schwartz et al., 1999). It is therefore increasingly difficult to discern whether the effect on cell function is directly related to Sr-release, the increased apatite precipitation on the surface of the material, or the altered release of other ions such as Ca^2+^, Si^4+^ and P^5+^, which have all been shown to positively affect bone cells (Habibovic and Barralet, 2011). Indeed, as Sr incorporation increases the entire material network is also altered, changing the bioactivity of the glass and the dissolution rates of Si, P and *Ca.*

#### 5.1.2 Ionic dissolution products

Ionic dissolution studies on bone implant materials help us to separate the effects of cell-surface contact from the effects of released ions. Extracts are produced by immersing materials in cell culture media; however, it is difficult to compare these studies because different materials are utilized with varying immersion times, producing different final Sr concentrations. One study by Zhang et al. (2011) produced extracts from HA and CaSi glass with increasing Sr incorporation by immersing them in DMEM for 1 day. They subsequently treated MG-63 cells (immortalized human osteosarcoma cells) with these extracts and measured ALP expression at different time points. What is most intriguing about their findings is that ALP expression is not linearly related to Sr concentration. For example, extracts produced from synthetic HA containing 10 mol% of Sr performed better at all time points than 40 and 100 mol% Sr-HA. These extracts contained 14 ppm of Sr compared to 35 ppm and 50 ppm, making it appear that the dose of 35 ppm is not effective at increasing ALP expression. Alternatively, they found that extracts from Sr-CaSi ceramics containing 5, 10 and 20 mol% Sr all performed better than controls at 3 and 7 days in terms of ALP expression. These extracts contained 32, 39 and 94 ppm of Sr. The effects are not completely dependent on Sr release because as Sr incorporation increases, the concentration of Ca, P and Si are also changing. Potentially, Sr interacts with other ions to enhance gene expression, or rather, when certain ions reach specific concentrations their effect dictates the cellular response over Sr’s effect.

#### 5.1.3 Sr interactions with Ca, Si and pH

As mentioned in a previous section, Sr and Ca combined have a greater effect on the production of inositol phosphate (IP), a second messenger involved in osteoblast replication and differentiation, than the individual action of either ion (Chattopadhyay et al., 2007). This effect has been shown to be mediated in part by the CaSR. One study similarly found that MC3T3-E1 pre-osteoblast cells presented with reduced mineralization and ALP expression after 8 days of culture with 1 mM of Sr in standard cell culture media (1.8 mM of Ca) (Xie et al., 2018). However, when cells were cultured with media supplemented with Ca (9 mM), the cells had enhanced bone cell function compared to controls at the same Sr dose. This may explain the results by Wornham et al. (2014), since they utilized standard Ca concentrations in their experiment, and similarly found ALP and nodule formation to be reduced by Sr treatment. This experiment sheds light on the importance of the microenvironment, since bone has higher Ca levels than plasma. It also suggests that the dual action of Ca and Sr from ion-leaching biomaterials may interact to enhance bone regeneration at the implant-bone interface.

Mao et al. (2017) sought to determine whether Si and Sr released from CaSi bioceramics had a synergistic effect to enhance osteogenesis and supress osteoclastogensis. Extracts from Sr-CaSi performed significantly better than extracts from tricalcium phosphate (TCP) in terms of ALP, OPG and OC expression in MSC's derived from ovariectomized rats. In a separate experiment, Mao et al. treated cells with Si and Sr ions alone or in combination, utilizing the same concentrations released in extracts. They found that while both ions individually had a stimulatory effect on ALP expression, Si had a stronger effect, and the combination of Si and Sr ions had the greatest effect. Similarly, both ions together had the greatest effect to inhibit osteoclastogenesis, but Sr alone had a stronger effect than Si alone. The experimenters concluded that while the two ions effect cell function to differing degrees, they synergize to intensify the effect.

Various studies have found that Sr incorporation into Ca—Si glasses leads to a more alkaline surface pH, since Sr^2+^ is a more powerful alkali ion than Ca^2+^ and these glasses have increased dissolution rates (Fredholm et al., 2012; Liu et al., 2016). Osteoblast cells have enhanced differentiation and proliferation when cultured in media that is more alkaline, since the activity of the ALP enzyme peaks at a pH of 8.5 (Harada et al., 1986). On the other hand, an acidic pH (below 7.4) favours osteoclast differentiation and leads to increased osteoclast activity (Meghji et al., 2001). Moreover, metabolic alkalosis has been shown to stimulate osteoblast activity (Bushinsky, 1996), and therefore, it has been postulated that a relatively alkaline pH (above 7.4) in the bone microenvironment is favoured for bone formation. The surface pH of a bioactive material can be largely different from the bulk pH of the solution it is immersed in (Liu et al., 2016; Shen et al., 2011), and this may have significant effects for cells residing in this microenvironment very close to the surface of the material. One study found that when extracts from Sr-CaSi glass were adjusted to match the interfacial pH of the material (~8.8), osteoblast ALP expression and number were increased above the effects of extracts at 7.4 (Shen et al., 2012). This provides an interesting nuance to the effects of incorporating Sr into biomaterials, since not only are the dissolution characteristics and bioactivity altered but the surface pH as well, contributing to the enhanced expression of osteogenic markers.

### 5.2 *In vivo*

There are multiple studies that have compared the *in vivo* effects of Sr-modified biomaterials to Sr-free materials, assessing osseointegration, healing and new bone formation at the bone-implant interface after implantation. Sr-enriched materials consistently perform better in these categories than similar materials lacking Sr, as shown in a systematic review by Neves et al. (2017). Their analysis found that out of 25 articles, 23 reported some kind of improvement in the Sr group, while no studies observed a decrease in bone formation compared to controls. Given the positive results from *in vitro t*esting, the local delivery of Sr into bone should enhance bone formation and reduce bone resorption around an implant, while mitigating the negative effects of systemic administration, such as increased risk of myocardial infarction (Abrahamsen et al., 2014; Cooper et al., 2014).

#### 5.2.1 Bone formation

Sr-doped CaSi ceramics (Liu et al., 2016; Lin et al., 2013), HA's (Wong et al., 2004) and CaP cements (Baier, 2006; Thormann et al., 2013) have been shown to promote new bone formation *in vivo* when implanted into a bone defect. However as discussed in the previous section, the effect of local Sr^2+^ release is difficult to assess since many other aspects of the material are enhanced. However, local Sr^2+^ release cannot be discounted as an important stimulator of new bone formation because relevant doses are present in the bone micro-environment surrounding the implant. An injectable Sr-HA containing bioactive bone cement was found to stimulate active bone formation and remodelling on its surface *in vivo*, resulting in increased osteoid and new bone formation 6 months after it was implanted into the iliac crest of rabbits (Wong et al., 2004). Energy-dispersive X-ray (EDX) microanalysis revealed that Sr was present on the surface of the Sr-HA bone cement as well as in the microenvironment between new bone and the implant. Similarly, while no Sr was found in the new bone formed around a Sr-CaSi glass, EDX analysis found that Sr was detected at the bone-implant interface, in the layer of apatite which joined the implant to the newly formed bone (Gorustovich et al., 2010). Thormann et al. utilized time-of-flight secondary ion mass spectrometry (*TOF*-*SIMS*) to observe the *in vivo* release of Sr from Sr-CaP cements, and found a high Sr concentration at the bone-implant interface, concluding that this release was most likely responsible for the enhanced bone formation they observed in the Sr-group (Thormann et al., 2013). In terms of clinical evidence, Sr-Si-HA's have been found to increase osteoblast activity significantly 12 months after implantation into the burr-holes of patients who underwent craniotomies (compared to bone at the craniotomy line) (*n* = 3) (Izci et al., 2013). Moreover, the ratio of osteoblastic activity at the biocermaic site/craniotomy site was the highest for Sr-Si-HA, compared to Si-HA and HA-wollastonite.

Otherwise inert materials coated with Sr, such as titanium implants coated with Sr—O, have also been shown to yield more favorable results than uncoated implants in rodent models (Offermanns et al., 2018; Andersen et al., 2013; Offermanns et al., 2015). These materials have been shown to release a steady dose of Sr^2+^, and have been shown to significantly accelerate bone ingrowth into the defect site, resulting in enhanced osseointegration, measured by % bone-implant contact (Offermanns et al., 2018). These studies provide strong evidence that local Sr^2+^ release alone is sufficient to increase bone formation *in vivo*, independent of Ca, P and Si release. There is a growing body of evidence that suggests that orally administered Sr/SrRan does not significantly stimulate bone formation *in vivo,* when subjects are receiving the normal dose and consuming a normal calcium diet. However, it's plausible that systemically delivered Sr does not reach high enough concentrations inside bone to produce a notable biological stimulation of bone formation *i.e.* osteoblast precursors/mature osteoblasts are not exposed to high enough doses to activate CaSR. Clinical trials for SrRan in some cases have noted a weak effect to reduce bone resorption, which is possibly because osteoclasts, located near blood/bone marrow are receiving a steady dose of Sr. The local release of Sr into a defect may expose osteoblasts to sufficient concentrations of Sr, leading to enhanced bone formation and osseointegration.

#### 5.2.2 Bone healing

Injury to the bone as a result of trauma, fracture or surgery initiates an immediate inflammatory reaction that is integral to bone healing. This process recruits cells to the site of injury in order to clear away debris and pathogens, which is followed by the activation of osteoprogenitor cells, angiogenesis and matrix formation (Marsell and Einhorn, 2011). The systemic administration of SrRan (625 mg/kg/day) in OVX rodents has been shown to significantly accelerate bone defect filling, improving cortical and trabecular microarchitecture in the defect site as well as material properties of the newly formed bone (Zacchetti et al., 2014; Li et al., 2010).

Strontium-modified biomaterials have been shown to elicit different patterns of bone healing after implantation compared to Sr-free materials (Henriques Lourenço et al., 2017; Cardemil et al., 2013), which suggests that Sr’s ability to enhance osseointegration/new bone formation may rely partially on its ability to effect inflammatory processes. Henriques Lorenço et al. utilized Sr-doped and Sr-free HA microspheres in an alginate hydrogel delivery system, and observed bone healing histologically after injection into a critical-sized metaphyseal defect in rats (Henriques Lourenço et al., 2017). They found that after 15 days, the Sr-group had increased inflammatory cell infiltration into the center of the defect. After 60 days, the Sr-group exhibited significantly enhanced granulation tissue formation, vascularization and osteoclast infiltration in the center of the defect compared to the Ca-group, and enhanced bone healing was accompanied by earlier bone formation and significantly thicker bone formed in the Sr-group. The experimenters concluded that a more rapid inflammatory response followed by accelerated bone remodelling (more osteoclasts present) resulted in increased bone formation. Cardemil et al. observed bone healing in rats implanted with either Sr-doped CaP granules or HA granules, and assessed inflammatory cytokine release and new bone formation in the tissue harvested from these defects after 6 and 28 days (Cardemil et al., 2013). They found that the two materials had completely different preferences for stimulating inflammatory cytokines such as tumor necrosis factor alpha (TNF-α) and interleukin-6 (IL-6). While Sr-particles significantly increased the expression of IL-6, HA-particles exclusively increased the expression of TNF-α. Interestingly, they also found that the new bone formed in the Sr group was mainly around the periphery of the defect while the bone formed in the HA group was concentrated in the center. Differences in the topological distribution of bone may be the result of different patterns of bone healing and may present an alternate mechanism to explain why Sr-materials perform better *in vivo* than their Sr-free counterparts.

## 6 Conclusion

The mechanism of action of Sr in bone remains elusive, even after decades of *in vivo*, *in vitro* and clinical studies. Even though Sr has been shown to activate the CaSR in osteoblast and osteoclast cells, experiments where a receptor is transfected to a cell line that does not endogenously express it presents certain limitations. This methodology entails overexpressing CaSR in a cell, which may produce an inordinately greater effect given the concentration of Sr used, that is not realistic to *in vivo* conditions. Additionally, it remains unclear how CaSR mediates the effects of Sr when administered to humans therapeutically, if it does at all. Clinical reports do not support CaSR's function in mediating Sr’s anabolic effects *in vivo*; however, it remains uncertain how the receptor is affected by the local delivery of Sr ions from Sr-modified biomaterials implanted directly in bone. The more recent studies on Sr do not support Sr’s anabolic effect, and instead support for the physiochemical mechanisms discussed. Sr treatment in clinical trials has been found to be responsible for increases in BMD, and while this may not reflect a true increase in bone tissue, these increases are correlated to reduced fracture risk. Increases in bone strength after Sr treatment are thought to be the result of sacrificial bond formation and possibly the stabilization of hydration levels, however, there is much less direct evidence for these mechanisms. Therefore, future studies will have to determine whether these mechanisms provide any explanation for Sr’s beneficial effect on fracture risk.

Finally, biomaterials enriched with Sr consistently perform better than their Sr-free counterparts both *in vitro* and *in vivo*. Despite changes to the solubility and ion release kinetics of Sr-doped biomaterials, some *in vivo* studies suggest that local Sr release alone is sufficient to promote bone formation in the absence of other osteogenic ions. Furthermore, accelerated bone healing has been observed with these materials, presenting an alternate mechanism to explain enhanced osseointegration. These findings are largely from animal studies, however, small case studies in humans have found Sr-doped materials to be biocompatible and useful for procedures such as vertebroplasty, kyphoplasty and craniotomy (Izci et al., 2013; Korovessis et al., 2018; Cheung et al., 2005). Nevertheless, studies with more patients are needed to strengthen these results and understand if the mechanisms discussed in this review pertain to humans. SrRan has been discontinued as an anti-osteoporotic drug since 2017; however, given the rise in Sr-enriched biomaterials in the last two decades, understanding Sr’s mechanism of action remains an important problem.

## Transparency document

Transparency document. Image 1
